# Intravesical Recurrence After Radical Nephroureterectomy of Upper Urinary Tract Urothelial Carcinoma: A Large Population-Based Investigation of Clinicopathologic Characteristics and Survival Outcomes

**DOI:** 10.3389/fsurg.2021.590448

**Published:** 2021-02-22

**Authors:** Jie Wu, Pei-Hang Xu, Wen-Jie Luo, Bo Dai, Yi-Jun Shen, Ding-Wei Ye, Yu-Chen Wang, Yi-Ping Zhu

**Affiliations:** ^1^Department of Urology, Fudan University Shanghai Cancer Center, Shanghai, China; ^2^Department of Oncology, Shanghai Medical College, Fudan University, Shanghai, China

**Keywords:** upper urinary tract urothelial carcinoma, intravesical recurrence, SEER, surgery, survival

## Abstract

**Background:** Of patients with upper urinary tract urothelial carcinoma (UTUC), 22–47% developed bladder recurrence after radical nephroureterectomy. Furthermore, the effect of surgery for UTUC-bladder cancer (BC) has not been well validated. The aim of this study was to assess the impact of standard primary BC surgical strategy on survival of patients diagnosed with UTUC-BC.

**Patients and Methods:** A total of 676 UTUC-BC patients and 197,753 primary BC patients diagnosed from 2004 to 2016, were identified based on the SEER database. The Kaplan-Meier method and the Fine and Gray competing risks analysis were performed to assess overall survival (OS) and cancer-specific mortality (CSM). Multivariate Cox regression model and competing risks regression model were used to identify independent risk factors. Propensity score matching (PSM) was also performed to adjust potential confounding factors.

**Results:** The baseline characteristics and survival outcomes of the two BC patient cohorts are quite different. For UTUC-BC patients, no significant difference in OS (NMIBC: *p* = 0.88; MIBC: *p* = 0.98) or cumulative incidence of CSM (NMIBC: *p* = 0.12; MIBC: *p* = 0.96) were noted for various surgical procedures. Local tumor treatment and partial cystectomy for UTUC-NMIBC patients produced lower 1-year (6.1%) and 3-year CSM (16.2%). Radical cystectomy for UTUC-MIBC patients produced lower 1-year (11.8%) but higher 3-year CSM (62.7%). After PSM for covariates, UTUC-BC patients still had a worse prognosis after surgery compared with primary BC patients. Based on regression models, older age, advanced T stage, N positive disease, M positive disease, and shorter interval between UTUC and BC were identified as independent risk factors for UTUC-BC patients.

**Conclusion:** Standard primary BC surgical strategy did not provide significant survival benefit for UTUC-BC patients. Compared with primary BC patients, UTUC-BC patients had a worse prognosis after surgery, suggesting that current primary BC surgical guidelines are not entirely appropriate for UTUC-BC patients. Our findings underscore the continued importance and need for better prognosis and improved guidelines for management of UTUC-BC patients.

## Introduction

Urothelial carcinomas (UCs) are the fourth most common malignancies in the world, representing a spectrum of diseases with a variety of prognoses (1). Upper urinary tract urothelial carcinomas (UTUCs) comprise ureter cancer and renal pelvis cancer, accounting for ~5–10% of urothelial carcinomas (2). Approximately 60% of UTUCs are invasive at first diagnosis and radical nephroureterectomy (RNU) with bladder cuff excision is the standard treatment for high-risk UTUC. For UTUC patients, 22–47% develop bladder recurrence after RNU (3–5). Therefore, bladder recurrence monitoring is essential UTUC management.

Although a number of studies have investigated the risk of intravesical recurrence (IVR) after RNU for UTUC, there are no large scale investigations of treatment strategies for this secondary bladder cancer (BC) (2, 6–8). Currently, the disease management of patients with IVR after RNU for UTUC (UTUC-BC) is based on the guidelines for primary BC. For non–muscle-invasive bladder cancer (NMIBC), transurethral resection of the bladder tumor (TURBT) remains the initial management choice. For muscle-invasive bladder cancer (MIBC), radical cystectomy (RC) is a commonly used curative treatment (9, 10). However, there are little data demonstrating improved survival for UTUC-BC patients treated by this therapy.

The aim of this study was to examine the impact of standard primary bladder cancer surgical strategy on survival of patients diagnosed with UTUC-BC using a large population-based cancer database.

## Materials and Methods

### Selection of Patient Cohort

The Surveillance, Epidemiology and End Results (SEER) database of the National Cancer Institute was used to conduct this retrospective study. UTUC cases included ureter cancer and renal pelvis cancer that were identified by the International Classification of Diseases-O-3 (ICD-O-3) codes C64.9, C65.9, and C66.9 from January 2004 to December 2016. Cases of transitional cell urothelial carcinoma were identified by ICD-O-3 code 8120-8139. Only patients who met the following criteria were included: (1) upper urinary tract as the first primary site; (2) RNU (surgical codes 40 or 50) was performed for UTUC; (3) bladder recurrence after RNU; (4) survival time ≥1 month; and (5) adequate tumor data were provided. Exclusion criteria were as follows: (1) a history of primary BC or a subsequent UTUC; (2) incomplete follow-up information; (3) metastatic cancer at the time of diagnosis of UTUC. A total of 676 UTUC patients who developed IVR after RNU (UTUC-BC) were included in the analysis with 197,753 primary transitional cell bladder carcinoma patients for comparison. Data were extracted from the SEER database using SEER^*^Stat Software (version 8.3.6).

### Data Collection and Variable Definition

Collected demographic variables included race, gender, marital status at IVR, age at IVR, and age at diagnosis of UTUC. Collected clinical variables included the Derived SEER Combined TNM system for cases diagnosed in 2016, the American Joint Commission on Cancer (AJCC) 6th Edition Staging for cases diagnosed from 2004 to 2015, tumor size, grade, tumor primary site of BC and UTUC. Collected therapy and follow-up variables included type of surgical procedure, radiation, chemotherapy, survival months, and vital status.

We restaged selected cases (2004–2015) according to the AJCC 8th Edition TNM system with the cases that could not be staged precisely referred to as Tx, Nx, or Mx. Surgical codes 50 (simple/total/complete cystectomy), 60 (complete cystectomy with reconstruction), 70 (pelvic exenteration, NOS), and 80 (cystectomy, NOS) for bladder cancer were merged and collectively defined as “radical cystectomy.” Surgical codes 10 (local tumor destruction, NOS) and 20 (local tumor excision, NOS) were combined and defined as “local tumor treatment.” The overall survival (OS) months for UTUC-BC were defined as the time from diagnosis of IVR to death or last follow-up, with patients still alive censored at the last follow-up. For cancer-specific mortality (CSM), deaths not due to bladder cancer or UTUC were considered as competing risks.

### Statistical Analysis

Demographic characteristics were summarized by count and percentage for categorical variables. Pearson's chi-square and Fisher's exact test were performed to compare the distribution of categorical data. Survival analysis was performed using the Kaplan-Meier method with *p*-values determined by log-rank test. Cumulative incidence function as well as Fine and Gray competing risk analysis were used to evaluate CSM (11, 12). Propensity-score matching (PSM) based on the nearest-neighbor matching principle was performed to adjust the potential differences in the backgrounds of the two groups. Multivariate Cox regression model and competing risk regression model were utilized to identify independent risk factors to predict overall survival and cancer-specific mortality of UTUC-BC patients. All statistical assessments were evaluated at a two-sided *p*-value of 0.05. All analyses were conducted using R software 3.5.2 (R Foundation for Statistical Computing, Vienna, Austria). The “survival” and “survminer” packages were used to perform survival analysis. The “cmprsk” package was used to perform competing risks analysis. The “maxstat” package was used to determine the cut-off values for continuity variables. The “mice” package was used to perform multivariate imputation.

## Results

### Demographic and Clinical Characteristics of UTUC-BC and Primary BC Patients

A total of 676 UTUC-BC patients and 197,753 primary BC patients were identified. The demographic and clinical characteristics of the two patient cohorts are listed in Tables 1, 2. Generally, the baseline characteristics of the two BC patient cohorts are quite different. For the UTUC-BC patient cohort, the majority of patients were white (595, 88.0%), male (397, 58.7%), with stage 0 (407, 60.2%) or I (172, 25.4%) BC TNM stage, and III/ IV grade (311, 46%). Median age at IVR and BC tumor size were 72.07 years and 24.36 mm, respectively. With regard to therapy, most patients underwent local tumor excision (571, 84.5%) including TURBT and did not receive radiation (658, 97.3%) or chemotherapy (590, 87.3%). Primary BC patients were more likely to be male (76.7%, *p* < 0.001), had larger tumor size (34.84 mm, *p* < 0.001), received radical cystectomy (8.4%, *p* < 0.001), and chemotherapy (20.6%, *p* < 0.001), compared with UTUC-BC patients. The primary BC patients also had higher TNM stage (*p* < 0.001). The primary site of tumor location was significantly different between UTUC-BC and primary BC patients (*p* < 0.001), primary BC patients were more likely to have tumors in the lateral wall of bladder (41,829, 21.2%). For the primary UTUC of UTUC-BC patients, the largest number of patients had renal pelvis cancer (505, 74.7%), III/ IV grade (457, 67.6%) and N0 stage (615, 91.0%). The median interval between UTUC and BC was 22.75 months.

**Table 1:** Demographic and clinical characteristics comparing patients with IVR after RNU for UTUC and primary bladder cancer.

		**IVR after RNU for UTUC (*****n*** **=** **676)**		**Primary bladder cancer (*****n*** **=** **197,753)**		
		**Count**	**%**	**Count**	**%**	***p-*value**
Race	White	595	88.0	176,291	89.1	<0.001^*^
	Black	30	4.4	10,793	5.5	
	Other	50	7.4	8,403	4.2	
	Unknow	1	0.1	2,266	1.1	
Gender	Male	397	58.7	151,383	76.6	<0.001^*^
	Female	279	41.3	46,370	23.4	
Marital status at IVR	Yes	351	51.9	117,085	59.2	<0.001^*^
	No	258	38.2	64,890	32.8	
	Unknown	67	9.9	15,778	8.0	
Tumor primary site of BC	Trigone of bladder	37	5.5	12,621	6.4	<0.001^*^
	Dome of bladder	27	4.0	6,380	3.2	
	Lateral wall of bladder	56	8.3	41,829	21.2	
	Anterior wall of bladder	10	1.5	4,032	2.0	
	Posterior wall of bladder	72	10.7	18,402	9.3	
	Bladder neck	51	7.5	5,739	2.9	
	Ureteric orifice	19	2.8	7,857	4.0	
	Urachus	0	0	35	0.0	
	Overlapping lesion of bladder	51	7.5	20,170	10.2	
	Bladder, NOS	353	52.2	80,688	40.8	
T stage of BC	T0	1	0.1	51	0.0	<0.001^*^
	Tis	53	7.8	9,993	5.0	
	Ta	354	52.4	100,298	50.7	
	T1	185	27.4	45,327	22.9	
	T2	44	6.5	25,221	12.8	
	T3	7	1.0	6,895	3.5	
	T4	14	2.1	5,985	3.0	
	Tx	18	2.7	3,974	2.0	
N stage of BC	N0	635	93.9	185,273	93.7	0.159
	N1	8	1.2	3,186	1.6	
	N2	2	0.3	2,057	1.0	
	N3	2	0.3	555	0.3	
	Nx	29	4.3	6,682	3.4	
M stage of BC	M0	663	98.1	192,355	97.3	0.506
	M1	12	1.8	4,930	2.5	
	Mx	1	0.1	468	0.2	
AJCC 8th stage of BC	0	407	60.2	110,099	55.7	<0.001^*^
	I	172	25.4	42,989	21.7	
	II	33	4.9	20,825	10.5	
	III	21	3.1	10,945	5.5	
	IV	13	1.9	5,730	2.9	
	Unknown	30	4.4	7,165	3.6	
Histopathological grade of BC	I	80	11.8	23,146	11.7	0.090
	II	157	23.2	49,601	25.1	
	III	128	18.9	31,529	15.9	
	IV	183	27.1	59,551	30.1	
	Unknown	128	18.9	33,926	17.2	
Type of surgical procedure	No surgery of primary site	60	8.9	12,236	6.2	<0.001^*^
	Local tumor destruction	8	1.2	1,671	0.8	
	Local tumor excision	571	84.5	164,206	83.0	
	Partial cystectomy	2	0.3	2,057	1.0	
	Radical cystectomy	29	4.3	16,623	8.4	
	Surgery NOS	3	0.4	367	0.2	
	Unknown	3	0.4	593	0.3	
Type of radiation	Beam radiation	16	2.4	7,961	4.0	0.061
	Other	2	0.3	521	0.3	
	Unknown	658	97.3	189,271	95.7	
Chemotherapy	Yes	86	12.7	406,93	20.6	<0.001^*^
	No/Unknown	590	87.3	157,060	79.4	
Tumor size, mm	Mean (SD)	24.36 (17.68)		34.84 (29.43)		<0.001^*^
Age at diagnosis of IVR or BC	Mean (SD)	72.07 (11.59)		71.10 (11.95)		0.700

IVR, intravesical recurrence; RNU, radical nephroureterectomy; UTUC, upper urinary tract urothelial carcinoma; BC, bladder cancer; AJCC, American Joint Committee on Cancer. The bold values and

* *indicated that p < 0.05*.

**Table 2:** Primary UTUC demographic and clinical information of the included patients with IVR after RNU for UTUC.

		**Count**	**%**
Primary site of UTUC	Renal pelvis	505	74.7
	Ureter	171	25.3
Grade of UTUC	I	35	5.2
	II	137	20.3
	III	221	32.7
	IV	236	34.9
	Unknown	47	7.0
T stage of UTUC	1	261	38.6
	2	113	19.7
	3	224	36.1
	4	26	3.8
	x	12	1.8
*N* stage of UTUC	0	615	91.0
	1	28	4.1
	2	11	1.6
	x	22	3.3
Interval between UTUC and BC (months)	Mean (SD)	22.75 (22.48)	
Age at diagnosis of UTUC	Mean (SD)	70.19 (11.60)	

*IVR, intravesical recurrence; RNU, radical nephroureterectomy; UTUC, upper urinary tract urothelial carcinoma; BC, bladder cancer*.

### Survival Comparison of UTUC-BC and Primary BC Patients

**Figure 1:**
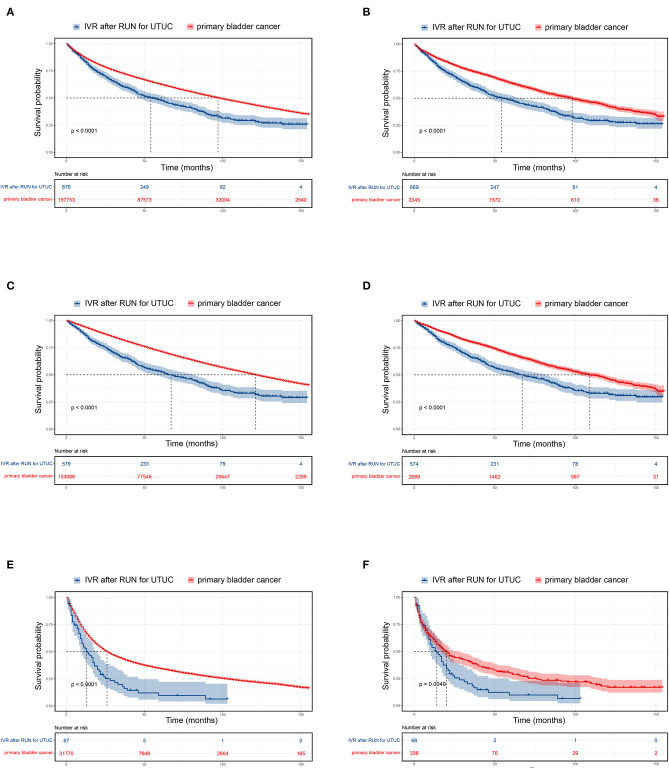
Kaplan–Meier curves and risk tables for UTUC-BC in comparison to primary BC. Entire patient cohort, **(A)** before propensity score matching (PSM); **(B)** after PSM. Non-muscle invasive BC, **(C)** before PSM; **(D)** after PSM. Muscle invasive and metastatic BC, **(E)** before PSM; **(F)** after PSM.

The median survival of UTUC-BC patients and primary BC patients was 54 and 97 months, respectively (Figure 1A, *p* < 0.001). For NMIBC, the median survival of UTUC-BC and primary BC patients was 67 and 112 months, respectively (Figure 1C, *p* < 0.001).For muscle invasive and metastatic BC, the median survival was 13 and 26 months, respectively (Figure 1E, *p* < 0.001). For each BC stage, overall survival probabilities were greater for primary BC vs. UTUC-BC (Table 3). The prognosis for UTUC-BC patients was significantly worse than for primary BC patients. Vital status and cause of death were stratified by stage for UTUC-BC and primary BC patients (Table 3). For NMIBC and MIBC, mortality due to BC was similar to UTUC-BC (23.6, 61.4%) and primary BC patients (19.4, 60.0%). However, mortality due to UTUC among UTUC-BC patients (20.9, 11.4%) was significantly greater than primary BC patients (1.2, 0.7%).

**Table 3:** Survival comparison between patients with UTUC-BC and patients with primary bladder cancer.

		**NMIBC (Stage 0–I)**		**MIBC (Stage II–III)**		**Metastatic cancer (Stage IV)**	
		**UTUC-BC**	**Primary BC**	**UTUC-BC**	**Primary BC**	**UTUC-BC**	**Primary BC**
Vital status	Alive	283 (48.9)	102,694 (67.1)	10 (18.5)	11,768 (37.0)	0 (0.0)	646 (11.3)
	Deceased	296 (51.1)	50,394 (32.9)	44 (81.5)	20,002 (63.0)	13 (100.0)	5,084 (88.7)
*p* value		<0.001^*^		0.007^*^		0.385	
Cause of death	Bladder cancer	70 (23.6)	9,874 (19.4)	27 (61.4)	11,988 (60.0)	6 (46.2)	3,886 (76.4)
	UTUC	62 (20.9)	589 (1.2)	5 (11.4)	140 (0.7)	4 (30.8)	56 (1.1)
	Other cancer	57 (19.3)	10,477 (20.8)	7 (15.9)	2,856 (14.3)	0 (0.0)	705 (13.9)
	Heart disease	36 (12.2)	10,514 (20.9)	2 (4.5)	1,750 (8.7)	0 (0.0)	149 (2.9)
	Other causes	71 (24.0)	19,529 (38.8)	3 (6.8)	3,268 (16.3)	3 (23.1)	288 (5.7)
*p* value		<0.001^*^		<0.001^*^		<0.001^*^	
Overall survival probabilities (%)	6-month survival	93.4 (91.4–95.4)	96.7 (96.7–96.8)	77.4 (67.0–90.0)	82.3 (81.9–82.8)	NA	49.0 (47.7–50.3)
	1-year survival	86.4 (83.6–89.2)	93.6 (93.5–93.8)	58.1 (46.1–73.1)	68.1 (67.5–68.6)	NA	28.1 (26.9–29.3)
	3-year survival	65.6 (61.6–70.0)	81.8 (81.6–82.0)	20.5 (11.6–36.0)	43.2 (42.7–43.8)	NA	8.0 (7.2–8.8)
	5-year survival	52.8 (48.5–57.5)	71.4 (71.1–71.6)	12.7 (5.8–28.2)	34.2 (33.6–34.8)	NA	5.0 (4.3–5.7)
Median survival months (95% CI)	67 (56–81)	121 (119–122)	17 (11–22)	26 (26–27)	NA	6 (6–7)	

UTUC-BC, bladder recurrence after radical nephroureterectomy of upper urinary tract urothelial carcinoma; NMIBC, non-muscle invasive bladder cancer; MIBC, muscle invasive bladder cancer; NA, not available. The bold values and

* *indicated that p < 0.05*.

### Overall Survival Analysis of UTUC-BC Patients Undergoing Different Surgical Procedures

**Figure 2:**
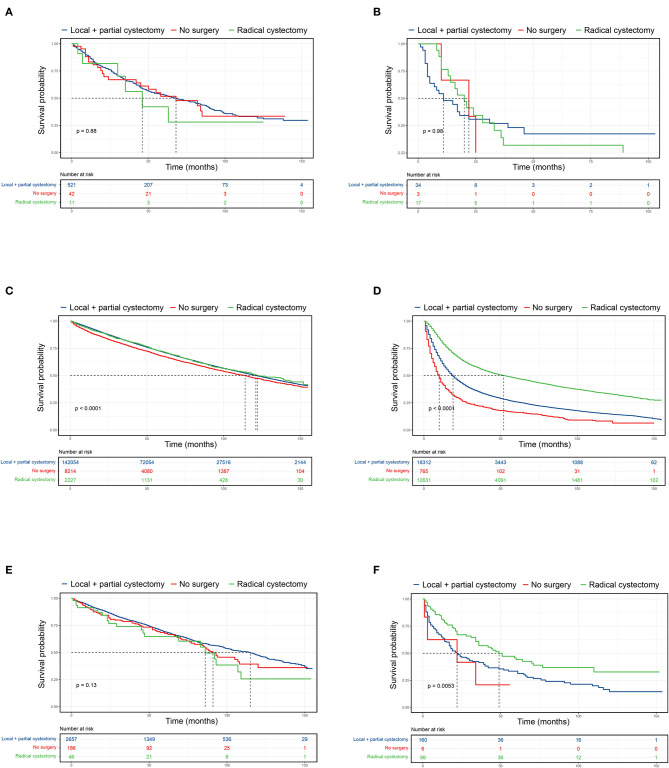
Kaplan-Meier curves comparing surgical benefits for UTUC-BC and primary BC. **(A)** OS of non-muscle invasive UTUC-BC. **(B)** OS of muscle invasive UTUC-BC. **(C)** OS of non-muscle invasive primary BC. **(D)** OS of muscle invasive primary BC. **(E)** OS of non-muscle invasive primary BC after PSM. **(F)** OS of muscle invasive primary BC after PSM.

Surgical procedures for UTUC-BC and primary BC patients were assessed (Table 4). For NMIBC of both BC patient cohorts, no survival difference based on surgical treatment was found (Figures 2A,C). However, UTUC-BC patients who received radical cystectomy had poorer outcomes, with an overall survival probability of 81.8% at 1 year and 56.1% at 3 years. Primary BC patients had significantly lower survival probability when surgery of the primary site was not performed (***p*** **<** **0.001**), with the shortest median survival (114 months). For UTUC-MIBC patients, radical cystectomy did not result in significantly greater survival compared to patients who received local procedures or partial cystectomy (*p* = 0.98), with an overall survival probability of 76.5% at 1 year and 13.7% at 3 years, but did have longer median survival (20 months) (Figure 2B). There was a significant difference in the survival of primary MIBC patients (***p*** **<** **0.001**). Patients receiving radical cystectomy had significantly longer median survival (52 months) than those receiving local procedures or partial cystectomy (19 months) (Figure 2D).

**Table 4:** Survival comparison between UTUC-BC and primary BC patients stratified by surgical procedure.

		**n**	**Median Survival (month)**	**1-yr OS, %**	**3-yr OS, %**	**1-yr!!break CSM, %**	**3-yr!!break CSM, %**
Non-muscle invasive UTUC-BC	No surgery of primary site	42	68 (45–NA)	83.2 (72.6–95.4)	66.9 (53.6–83.6)	12.0	22.9
	Local tumor treatment or Partial cystectomy	521	68 (54–84)	86.6 (83.7–89.6)	65.6 (61.4–70.1)	6.1	16.2
	Radical cystectomy	11	46 (30–NA)	81.8 (61.9–100)	56.1 (30.8–100)	18.2	43.9
Non-muscle invasive primary BC	No surgery of primary site	2,946	114 (108–118)	89.8 (89.1–90.4)	78.0 (77.1–79.0)	3.0	5.8
	Local tumor treatment or Partial cystectomy	142,054	121 (120–122)	93.9 (93.8–94.0)	82.0 (81.8–82.2)	1.6	4.5
	Radical cystectomy	712	122 (115–140)	92.9 (91.9–94.0)	82.0 (80.3–83.8)	3.6	9.4
Non-muscle invasive primary BC after PSM	No surgery of primary site	186	91 (79–120)	89.8 (85.5–94.4)	78.5 (72.5–85.0)		
	Local tumor treatment or Partial cystectomy	2,657	115 (103–121)	92.9 (91.9–93.9)	80.2 (78.6–81.9)		
	Radical cystectomy	46	86 (69–NA)	91.3 (83.5–99.8)	74.0 (61.8–88.6)		
Muscle invasive UTUC-BC	No surgery of primary site	3	NA	NA	NA	NA	NA
	Local tumor treatment or Partial cystectomy	34	11 (5–31)	47.8 (33.3–68.6)	26.9 (14.9–48.7)	39.7	53.8
	Radical cystectomy	17	20 (14–36)	76.5 (58.7–99.5)	13.7 (3.9–48.7)	11.8	62.7
Muscle invasive primary BC	No surgery of primary site	765	10 (9–11)	42.2 (38.8–46.0)	22.0 (19.1–25.3)	36.1	48.6
	Local tumor treatment or Partial cystectomy	18,312	19 (18–19)	61.0 (60.2–61.7)	34.9 (34.2–35.6)	25.9	41.8
	Radical cystectomy	12,631	52 (49–56)	79.9 (79.2–80.6)	56.7 (55.8–57.6)	13.7	30.1
Muscle invasive primary BC after PSM	No surgery of primary site	6	NA	NA	NA		
	Local tumor treatment or Partial cystectomy	160	22 (17–39)	65.0 (57.9–72.9)	41.5 (34.2–50.4)		
	Radical cystectomy	99	49 (36–77)	81.4 (74.0–89.5)	56.9 (47.4–68.3)		

*UTUC-BC, bladder recurrence after radical nephroureterectomy of upper urinary tract urothelial carcinoma; BC, bladder cancer; OS, overall survival; CSM, cancer-specific mortality; NA, not available; PSM, propensity score matching*.

### Cancer-Specific Mortality Analysis of UTUC-BC Patients Undergoing Different Surgical Procedures

**Figure 3:**
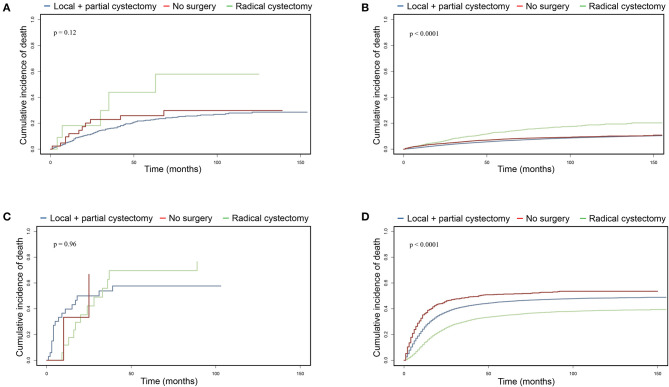
Cumulative incidence of cancer-specific mortality comparing surgical benefits for UTUC-BC and primary BC based on competing risks analysis. **(A)** CSM of non-muscle invasive UTUC-BC. **(B)** CSM of non-muscle invasive primary BC. **(C)** CSM of muscle invasive UTUC-BC. **(D)** CSM of muscle invasive primary BC.

To further investigate surgical benefits for UTUC-BC patients, we performed cancer-specific mortality analysis using Fine and Gray competing risks analysis. For UTUC-NMIBC patients, 132 of 296 deaths were due to UTUC or BC. No significant difference in cumulative incidence of CSM were noted among surgical procedures (Figure 3A, *p* = 0.12). Patients who received local procedures or partial cystectomy had a probability of death due to UTUC or BC of 6.1% at 1 year and 16.2% at 3 years. For UTUC-MIBC patients, 32 of 44 deaths were due to UTUC or BC. There was no significant difference in cumulative incidence of CSM for surgical procedures (Figure 3C, *p* = 0.96). Patients who received radical cystectomy had a probability of death due to UTUC or BC of 11.8% at 1 year and 62.7% at 3 years. For primary NMIBC patients, 10,463 of 50,394 deaths were due to UTUC or BC. Patients who underwent radical cystectomy had the worst prognosis (***p*** **<** **0.001**), with a probability of death due to UTUC or BC of 3.6% at 1 year and 9.4% at 3 years (Figure 3B). For primary MIBC patients, 12,128 of 20,002 deaths were due to UTUC or BC. Patients who underwent radical cystectomy had the best prognosis (***p*** **<** **0.001**), with a probability of death due to UTUC or BC of 13.7% at 1 year and 30.1% at 3 years (Figure 3D).

### PSM Analysis of UTUC-BC and Primary BC

After PSM for covariates, significantly difference in the baseline characteristics of UTUC-BC and BC demonstrated an even distribution (Supplementary Table 1). The prognosis for UTUC-BC patients was significantly worse than for primary BC patients (Figures 1B,D,F and Supplementary Table 2). After the adjustment, no survival difference based on surgical treatment was found for primary NMIBC patients (Figure 2E, *p* = 0.13), while MIBC patients who underwent radical cystectomy had the best prognosis (Figure 2F, ***p*** **=** **0.005**). UTUC-BC patients still had a worse prognosis after surgery compared with primary BC patients (Table 4).

### Multivariate Cox Regression Analysis and Multivariable Competing Risks Regression Analysis

**Figure 4:**
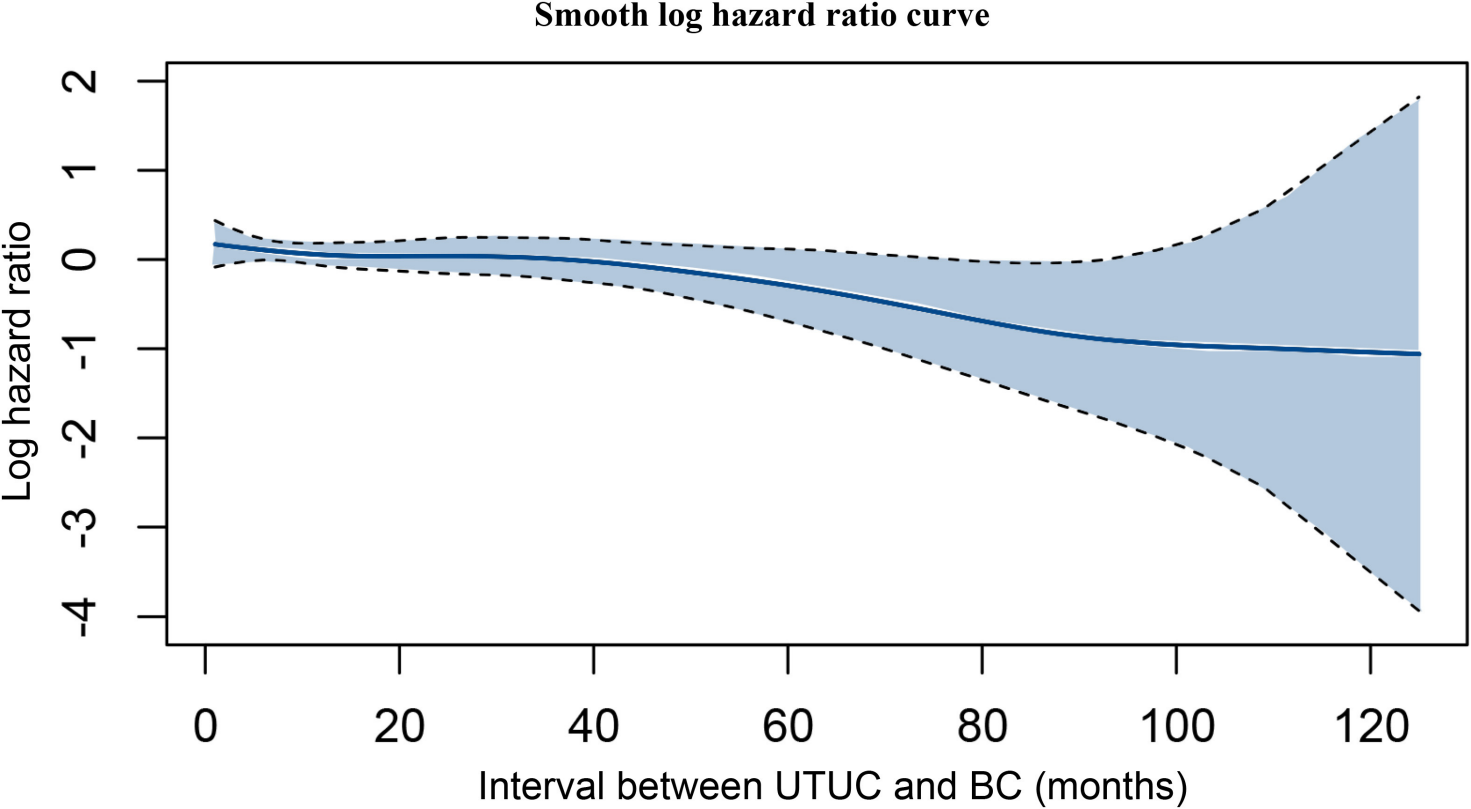
Hazard ratio curve of interval between UTUC and BC.

Based on the multivariate Cox regression model, older age, advanced T stage, N positive disease, M positive disease, shorter interval between UTUC and BC, and higher grade of BC were identified as independent risk factors associated with poorer OS (Table 5). Using a multivariable competing risks regression model, independent factors that associated with increased CSM for UTUC-BC patients were validated, including older age, advanced T stage, N positive disease, M positive disease, and shorter interval between UTUC and BC. Based on smooth hazard ratio (HR) analysis, the relationship among interval between UTUC and BC, and OS of UTUC-BC patients was assessed. The log HR curve showed a tendency for decline, indicating poorer prognosis for UTUC patients who developed earlier IVR (Figure 4).

**Table 5:** Multivariate Cox regression analysis for prediction of overall survival and multivariable competing risks regression analysis for prediction of cancer-specific mortality for UTUC-BC.

	**Overall survival**			**Cancer-specific mortality**		
	**HR**	**95%CI**	***p*-value**	**SHR**	**95%CI**	***p*-value**
**Age**						
<73	Ref		<0.001*	Ref		<0.001*
≥73	2.039	1.642–2.530		2.032	1.654–2.548	
**T stage of BC**						
Ta/Tis/T1	Ref		0.013*	Ref		<0.010*
T2–4	4.577	1.385–15.127		2.628	1.916–3.944	
***N*** **stage of BC**						
N0	Ref		0.020*	Ref		0.033*
N1–N3	2.015	1.118–3.632		1.818	1.046–2.957	
**M stage of BC**						
M0	Ref		<0.001*	Ref		<0.001*
M1	4.708	1.969–11.257		3.295	2.106–6.056	
**Interval between UTUC and BC**						
≥48	Ref		0.006*	Ref		0.003*
<48	1.780	1.176–2.695		1.774	1.225–2.647	
**Grade of BC**						
I/ II	Ref		0.049*	Ref		0.069
III/ IV	1.274	1.001–1.620		1.260	1.023–1.661	

*UTUC-BC, bladder recurrence after radical nephroureterectomy of upper urinary tract urothelial carcinoma; BC, bladder cancer; HR, hazard ratio; SHR, subdistribution hazard ratio; CI, confidence interval; Ref, reference*.

## Discussion

UTUC is an aggressive malignancy, comprising 5–10% of all urothelial carcinomas. Although diagnosis and management of UTUC have undergone significant improvements, a percentage of UTUC patients treated with RNU go on to develop intravesical recurrence (1, 3). The pathogenesis of IVR after RNU for UTUC still remains unclear. Several studies have assessed clinicopathologic factors linked to IVR and several risk factors including T stage, multifocality, and the use of diagnostic ureteroscopy have been identified (6, 13). Management of subsequent bladder cancer after RNU for UTUC is identical to the current treatment strategy for any primary BC (3, 14). Based on NCCN and EAU guidelines for bladder cancer, TURBT is the standard treatment for NMIBC and radical cystectomy is the standard treatment for MIBC. However, due to the impact of the primary UTUC and the biological differences between these two distinct urothelium-derived malignancies, multicenter studies need to be conducted to validate this management strategy (15). Miyake et al. reviewed 256 UTUC-NMIBC patients, and 75 of these patients received intravesical BCG after TURBT. The results demonstrated that UTUC-NMIBC patients to have a worse prognosis after intravesical BCG compared to primary NMIBC, indicating a poor response to BCG (16). Moreover, evidence for the benefits of surgery for UTUC-BC patients remains insufficient.

In this study based on the SEER program database, we identified UTUC-BC to be more commonly associated with lower TNM stage, smaller tumor size, female gender, and less radical cystectomy compared to primary BC. The UTUC patients underwent stringent follow-up for over 5 years after RNU, with surveillance regimens mainly based on white light cystoscopy, with association probably attributable to the early stage and small size of the IVR (3). Our studies also showed a poorer prognosis for UTUC-BC patients compared to primary BC. Notably, vital status and cause of death for UTUC-BC patients and primary BC patients differed significantly. The combined percentage of UTUC and BC deaths was greater among UTUC-BC patients. More importantly, results indicated that radical cystectomy or TURBT did not provide significant survival benefit for UTUC-NMIBC patients compared to no primary site surgery, although patients who received local tumor treatment or partial cystectomy had better 1- and 3-year OS, with lower 1- and 3-year CSM. Radical cystectomy did not improve survival of UTUC-MIBC patients, although the patients did have a longer median survival. These results indicate that current primary BC treatment guidelines are not entirely appropriate for UTUC-BC patients. There are several reasons why UTUC-BC patients may not benefit from standard primary BC treatment. First, UTUC is an aggressive neoplasm with a poorer prognosis than BC (3). Therefore, a history of UTUC would definitely influence the outcomes of patients diagnosed with secondary BC. Second, based on Table 1, the demographic and clinical characteristics of UTUC-BC and primary BC patients are quite different. Moreover, Yates et al. highlighted significant differences in the genetic and epigenetic backgrounds of UTUC-BC and primary BC patients (17). These differences may be partially attributable to different treatment effects for the two diseases.

Considering the significant difference between UTUC-BC and primary BC, we conducted PSM to adjust for potential differences in the backgrounds of the two groups. After PSM for covariates, UTUC-BC patients still had a worse prognosis after surgery compared with primary BC patients. Furthermore, we performed multivariate Cox regression analysis and competing risks regression analysis to identify independent risk factors that predict OS and CSM for secondary BC. Notably, except for well-recognized risk factors including older age and advanced tumor stage, shorter interval between UTUC and BC was also significantly associated with poorer OS and higher CSM, indicating that early developed IVR tended to be more aggressive. Hou et al., using the SEER database, developed a nomogram to predict cancer-specific survival (CSS) for UTUC-BC patients (18). However, that study did not assess surgical benefits for UTUC-BC patients and had several shortcomings. First, when performing CSS analysis, the authors defined the end point as death secondary to UTUC, which ignored death secondary to BC, significantly affecting the efficacy of the prognostic nomogram. Moreover, the authors defined patients who died secondary to other causes as censored data and used log-rank tests to access CSS, which would contribute to competing risk bias.

Despite several promising results, this registry-based study has unavoidable limitations. First, limitations of SEER-based studies include the absence of detail with regard to individual information for chemotherapy regimen and radiotherapy doses/fields, relevant information about decision-making process of UTUC-BC, and factors that may have bearing on intravesical recurrence rates such as the use of a ureteroscope and the smoking history of patients. Second, intravesical recurrence rates for UTUC patients after RNU based on the SEER database were ~8%, significantly lower than previously reported as 22–47% in the literature (3). Bias may also due to the limitations of any registry-based study. The UTUC-BC patients were only captured by the database if the bladder recurrence occurred in one of the SEER catchment areas. Third, the number of UTUC-MIBC cases was small, thus multicenter prospective studies are needed to validate relevant findings.

To our knowledge, this is the first large population-based study to investigate the survival of UTUC-BC patients stratified by surgical procedure. Despite the limitations of this study, our results highlight the significant differences between UTUC-BC and primary BC patients on demographics and clinical characteristics, and oncological outcomes. More importantly, our results suggest that current primary BC surgical guidelines may not be appropriate for UTUC-BC patients and identify shorter interval between UTUC and BC as an independent risk factor. Our findings underscore the continued importance and need for better prognosis and improved guidelines for management of UTUC-BC patients.

## Data Availability Statement

The original contributions presented in the study are included in the article/Supplementary Material, further inquiries can be directed to the corresponding author/s.

## Ethics Statement

The current study is based on the Surveillance, Epidemiology and End Results (SEER) database of the National Cancer Institute. Thus, ethical review and approval were not required for the study on human participants in accordance with the local legislation and institutional requirements. Written informed consent for participation was not required for this study in accordance with the national legislation and the institutional requirements.

## Author Contributions

JW: data collection, data analysis, and manuscript writing. P-HX: data analysis. W-JL: data collection. BD: manuscript editing. Y-JS and D-WY: project development. Y-CW: data analysis and manuscript editing. Y-PZ: project development and manuscript editing. All authors contributed to the article and approved the submitted version.

## Conflict of Interest

The authors declare that the research was conducted in the absence of any commercial or financial relationships that could be construed as a potential conflict of interest.
