# Supplementary material for: Intravesical Recurrence After Radical Nephroureterectomy of Upper Urinary Tract Urothelial Carcinoma: A Large Population-Based Investigation of Clinicopathologic Characteristics and Survival Outcomes
Source: Front Surg. 2021 Feb 22;8:590448. doi: 10.3389/fsurg.2021.590448 (PMC7938894; doi:10.3389/fsurg.2021.590448)
Supplement: Supplementary file 1 [file Table_1.pdf]

Supplementary Table 1. Demographic and clinical characteristics comparing patients with IVR after RNU for UTUC and primary bladder cancer after PSM.

		IVR after RNU for URUC (n=669)		Primary bladder cancer (n=3345)		
		Count	%	Count	%	*p* value
Race	White	588	87.9	2938	87.8	0.989
	Black	30	4.5	157	4.7	
	Other	50	7.5	243	7.3	
	Unknow	1	0.1	7	0.2	
Gender	Male	395	59.0	1922	57.5	0.475
	Female	274	41.0	1423	42.5	
Marital status at IVR	Yes	350	52.3	1730	51.7	0.795
	No	254	38.0	1310	39.2	
	Unknown	65	9.7	305	9.1	
Tumor primary site of BC	Trigone of bladder	37	5.5	186	5.6	0.992
	Dome of bladder	27	4.0	155	4.6	
	Lateral wall of bladder	56	8.4	284	8.5	
	Anterior wall of bladder	10	1.5	64	1.9	
	Posterior wall of bladder	71	10.6	361	10.8	
	Bladder neck	51	7.6	234	7.0	
	Ureteric orifice	19	2.8	87	2.6	
	Overlapping lesion of bladder	51	7.6	242	7.2	
	Bladder, NOS	347	51.0	1732	51.8	
T stage of BC	T0	0	0	3	0.1	0.849
	Tis	52	7.8	235	7.0	
	Ta	359	52.3	1712	51.2	
	T1	185	27.7	1004	30.0	
	T2	44	6.6	217	6.5	

	T3	7	1.0	39	1.2	
	T4	14	2.1	67	2.0	
	Tx	17	2.5	68	2.0	
N stage of BC	N0	630	94.2	3158	94.4	0.938
	N1	8	1.2	46	1.4	
	N2	2	0.3	11	0.3	
	N3	2	0.3	7	0.2	
	Nx	27	4.0	123	3.7	
M stage of BC	M0	657	98.2	3270	97.8	0.869
	M1	11	1.6	67	2.0	
	Mx	1	0.1	8	0.2	
AJCC 8th stage of BC	0	402	60.1	1974	58.2	0.782
	I	172	25.7	942	28.2	
	II	33	4.9	165	4.9	
	III	21	3.1	100	3.0	
	IV	12	1.8	71	2.1	
	Unknown	29	4.3	120	3.6	
Histopathological grade of BC	I	78	11.7	383	11.4	0.988
	II	156	23.3	783	23.4	
	III	126	18.8	603	18.0	
	IV	183	27.4	935	28.0	
	Unknown	126	18.8	641	19.2	
Type of surgical procedure	No surgery of primary site	59	8.8	250	7.5	0.443
	Local tumor treatment or Partial cystectomy	581	86.8	2934	87.7	
	Radical cystectomy	29	4.3	161	4.8	

Type of radiation	Beam radiation	16	2.4	72	2.2	0.878
	Other	1	0.1	5	0.1	
	Unknown	652	97.5	3268	97.7	
Chemotherapy	Yes	86	12.9	429	12.8	1.000
	No/Unknown	583	87.1	2916	87.2	
Tumor size, mm	< 30	144	21.5	713	21.3	0.966
	⩾30	56	8.4	290	8.7	
	Unknown	469	70.1	2342	70.0	
Age at diagnosis of IVR or BC	Mean (SD)	72.02 (11.62)		72.22 (11,71)		0.843

IVR: intravesical recurrence; RNU: radical nephroureterectomy; UTUC: upper urinary tract urothelial carcinoma; BC: bladder cancer; PSM: propensity score matching; AJCC: American Joint Committee on Cancer
