# Supplementary material for: Intravesical Recurrence After Radical Nephroureterectomy of Upper Urinary Tract Urothelial Carcinoma: A Large Population-Based Investigation of Clinicopathologic Characteristics and Survival Outcomes
Source: Front Surg. 2021 Feb 22;8:590448. doi: 10.3389/fsurg.2021.590448 (PMC7938894; doi:10.3389/fsurg.2021.590448)
Supplement: Supplementary file 2 [file Table_2.pdf]

Supplementary Table 2. Survival comparison between patients with UTUC-BC and patients with primary bladder cancer after PSM.

		NMIBC (Stage 0-I)		MIBC (Stage II- III)		Metastatic cancer (Stage IV)	
		UTUC-BC	Primary BC	UTUC-BC	Primary BC	UTUC-BC	Primary BC
Vital status	Alive	281 (49.0)	1839 (63.7)	10 (18.5)	97 (36.6)	0 (0.0)	5 (7.0)
	Deceased	293 (51.0)	1050 (36.3)	44 (81.5)	168 (63.4)	12 (100.0)	66 (93.0)
p value		**< 0.001***		**0.016***		1.000	
Overall survival probabilities (%)	6-month survival	93.3 (91.3-95.4)	96.2 (95.5-96.9)	77.4 (67.0-89.5)	80.1 (75.4-85.1)	NA	40.9 (30.9-54.0)
	1-year survival	86.3 (83.4-89.2)	92.6 (91.7-93.6)	58.1 (46.1-73.1)	71.0 (65.7-76.8)	NA	24.1 (15.8-36.8)
	3-year survival	65.6 (61.6-69.8)	80.0 (78.5-81.6)	20.5 (11.6-35.9)	46.8 (40.8-53.6)	NA	8.0 (3.5-18.5)
	5-year survival	52.7 (48.3-57.4)	68.9 (67.0-70.8)	12.7 (5.8-28.2)	37.1 (31.1-44.1)	NA	NA
Median survival months (95% CI)		67 (55-82)	109 (102-118)	17 (11-22)	34 (22-44)	NA	5 (3-7)

UTUC-BC: bladder recurrence after radical nephroureterectomy of upper urinary tract urothelial carcinoma; PSM: propensity score matching; NMIBC: non-muscle invasive bladder cancer; MIBC: muscle invasive bladder cancer; NA: not available
